# Genome-Based Reclassification of Strain KIST612, Previously Classified as *Eubacterium limosum*, into a New Strain of *Eubacterium callanderi*

**DOI:** 10.4014/jmb.2304.04011

**Published:** 2023-05-18

**Authors:** Ji-Yeon Kim, Byeongchan Kang, Soyoung Oh, Yeji Gil, In-Geol Choi, In Seop Chang

**Affiliations:** 1 School of Earth Sciences and Environmental Engineering, Gwangju Institute of Science and Technology, Gwangju 61005, Republic of Korea; 2 Research Center for Innovative Energy and Carbon Optimized Synthesis for Chemicals (inn-ECOSysChem), Gwangju Institute of Science and Technology, Gwangju 61005, Republic of Korea; 3 Department of Biotechnology, College of Life Sciences and Biotechnology, Korea University, Seoul 02841, Republic of Korea

**Keywords:** *Eubacterium*, 16S rDNA phylogeny, housekeeping phylogeny, proteoma-core phylogeny, genome metrics, reclassification

## Abstract

The strain KIST612, initially identified as *E. limosum*, was a suspected member of *E. callanderi* due to differences in phenotype, genotype, and average nucleotide identity (ANI). Here, we found that *E. limosum* ATCC 8486^T^ and KIST612 are genetically different in their central metabolic pathways, such as that of carbon metabolism. Although 16S rDNA sequencing of KIST612 revealed high identity with *E. limosum* ATCC 8486^T^ (99.2%) and *E. callanderi* DSM 3662^T^ (99.8%), phylogenetic analysis of housekeeping genes and genome metrics clearly indicated that KIST612 belongs to *E. callanderi*. The phylogenies showed that KIST612 is closer to *E. callanderi* DSM 3662^T^ than to *E. limosum* ATCC 8486^T^. The ANI between KIST612 and *E. callanderi* DSM 3662^T^ was 99.8%, which was above the species cut-off of 96%, Meanwhile, the ANI value with *E. limosum* ATCC 8486^T^ was not significant, showing only 94.6%. The digital DNA-DNA hybridization (dDDH) results also supported the ANI values. The dDDH between KIST612 and *E. callanderi* DSM 3662^T^ was 98.4%, whereas between KIST612 and *E. limosum* ATCC 8486^T^, it was 57.8%, which is lower than the species cut-off of 70%. Based on these findings, we propose the reclassification of *E. limosum* KIST612 as *E. callanderi* KIST612.

## Introduction

Strain KIST612 is a rod-shaped, non-spore-forming gram-positive bacterium and one of the representative acetogens used for C1 gas upcycling. This strain was first isolated in an anaerobic digester in 1997 and later identified as *Eubacterium limosum* based on 16S rDNA identity comparison [1]. In an effort to understand and engineer the metabolism, the whole genome of KIST612 was fully sequenced in 2011 and deposited in the NCBI database (http://www.ncbi.nlm.nih.gov) as CP002273 (GenBank Accession No. GCF_000152245) [2]. In 2018, the NCBI automatically reclassified *E. limosum* KIST612 as *E. callanderi* KIST612, based on the average nucleotide identity (ANI) between GCF_000152245 and GCF_900142645, which was newly deposited in 2016. It could be supported that KIST612 did not belong to *E. limosum* due to the phenotype difference between KIST612 and *E. limosum* ATCC 8486^T^. Both strains were recognized as CO_2_ (with H2) and CO utilizers, with potential applications in large-scale industrial processes in the energy field. Studies accumulated over more than 20 years have demonstrated the higher CO tolerance of KIST612 in comparison with ATCC 8486 [1, 3]. In addition, a recent study indicated a metabolic difference between the two strains through butyrate production under H_2_/CO_2_ condition [4].

Despite the suspicion concerning the taxonomic classification of KIST612, a lack of evidence for reclassification has added confusion to the “official taxonomic name.” Indeed, since 2020, there have already been several instances where KIST612 was used alone or together with ‘*E. callanderi*’ [5-9]. The present study can provide clarification of the taxonomic position of KIST612 strain based on phylogenomic study. For this purpose, we analyzed the sequence identities between conserved genes (*i.e.*, 16S rDNA, *rpoB*, and *gyrA*) and the core-proteome of 34 strains. The metabolic pathways were then compared between three *Eubacterium* strains, *E. limosum* ATCC 8486^T^, *E. callanderi* DSM 3662^T^, and KIST612. Through these analyses, we propose reclassification of KIST612 as a new strain of *E. callanderi*.

## Materials and Methods

### Genome Collection

The genome sequences of 25 *Eubacterium* spp. and of reclassified strains from *Eubacterium* (*Amedibacillus dolichus* VPI C9-20T, *Anaerobutyricum hallii* VPI B4-27T, *An. soehngenii* DSM 17630T, *Collinsella aerofaciens* VPI 1003T, *Dorea formicigenerans* VPI C8-13T, *Faecalitalea contorta* VPI 0119T, *F. cylindroides* VPI 3654T, *Holdemanella biformis* VPI C17-5T, and *Lachnoanaerobaculum saburreum* VPI 11763T) were obtained from the NCBI database [10-16]. To facilitate phylogenetic analysis, strains were selected as type strains (Table 1).

### Phylogenetic Reconstruction

Sequences of 16S rDNA and housekeeping genes for phylogenetic analysis of *Eubacterium* spp. and their relatives were aligned using the MUSCLE algorithm in MEGA11 (Molecular Evolutionary Genetics Analysis version 11) [17, 18]. Each analysis was performed based on a maximum-likelihood phylogenetic tree and its bootstrap values were calculated from 1,000 replicates. The core-proteome phylogenetic analysis was performed based on the pan proteome from each organism’s genomic data through BPGA (Bacterial Pan Genome Analysis)[19]. The core-proteome phylogenetic analysis was performed based on the neighbor-joining phylogenetic tree algorithm in BPGA.

### Sequence Comparison

Analysis of the genomes of *Eubacterium* spp. and their relatives was performed using OrthoANIu (Average Nucleotide Identity by Orthology using USEARCH) and digital DNA-DNA hybridization (dDDH) prediction, which is capable of quickly analyzing the similarity of the entire DNA sequence. OrthoANIu was calculated using OAU (https://www.ezbiocloud.net/tools/orthoaniu) analyzed by USEARCH, and dDDH was estimated using GGDC (Genome Distance Calculator for Genomes, http://ggdc.dsmz.de/ggdc.php) [20-22]. We presented the results of OrthoANIu and dDDH in the form of a matrix to better visualize the genomic relationship between *Eubacterium* spp. and their relatives.

### Pathway Comparison

Comparison between the metabolic pathways of *E. limosum* ATCC 8486^T^, *E. callanderi* DSM 3662^T^, and strain KIST612 was performed using KEGG (Kyoto Encyclopedia of Genes and Genomes) database and BlastKOALA (Blast KEGG Orthology And Links Annotation) [23, 24]. The pathway analysis was visualized using iPath3 (http://pathways.embl.de) [25].

## Results and Discussion

### Taxonomic Position of Strain KIST612 Based on 16S rDNA, Housekeeping Genes, and Core-Proteome Phylogenies

**Fig. 1:**
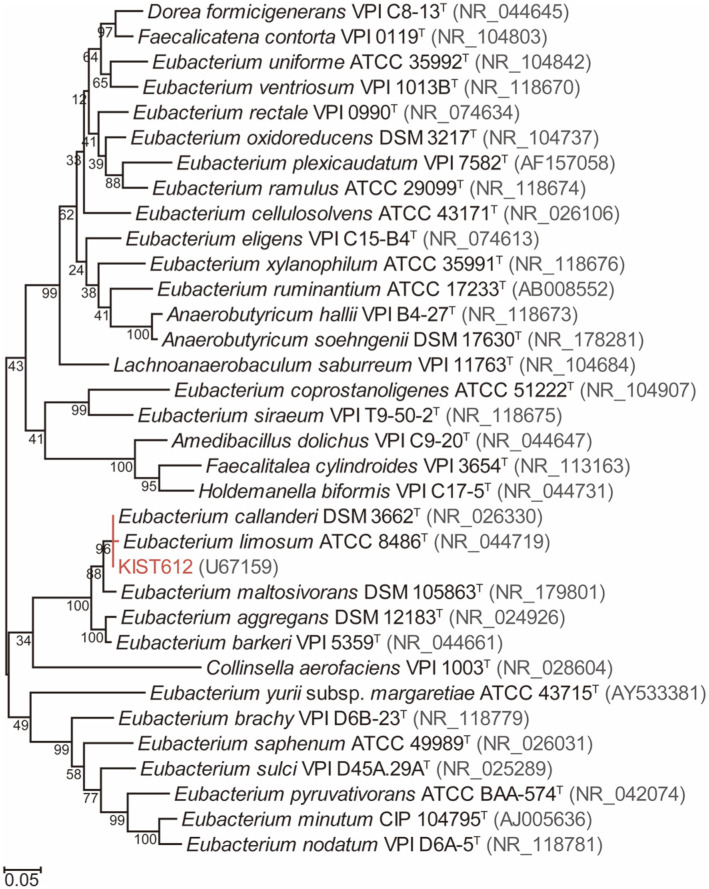
Maximum-likelihood phylogenetic tree of 16S rDNA sequences of *Eubacterium* spp. and their relatives. Bootstrap values were calculated from 1,000 replicates and are represented at each node. Accession numbers of 16S rDNA sequences are provided next to the species name. The bar means 0.05 nucleotide substitution per site.

**Fig. 2:**
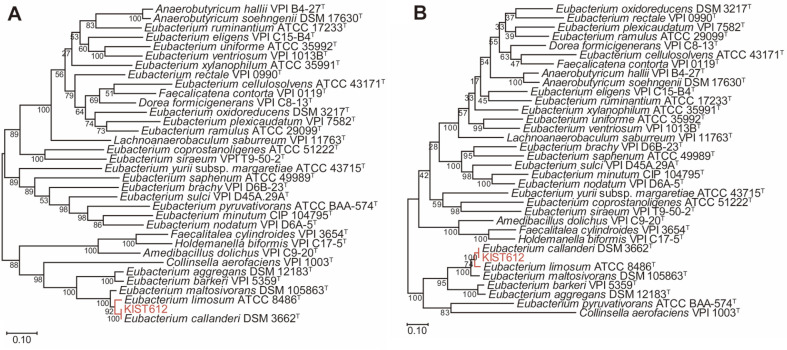
Maximum-likelihood phylogenetic tree of 2 housekeeping genes, (A) *rpoB* and (B) *gyrA*, of *Eubacterium* spp. and their relatives. Bootstrap values were calculated from 1,000 replicates and are represented at each node. The bar means 0.10 nucleotide substitution per site.

**Fig. 3:**
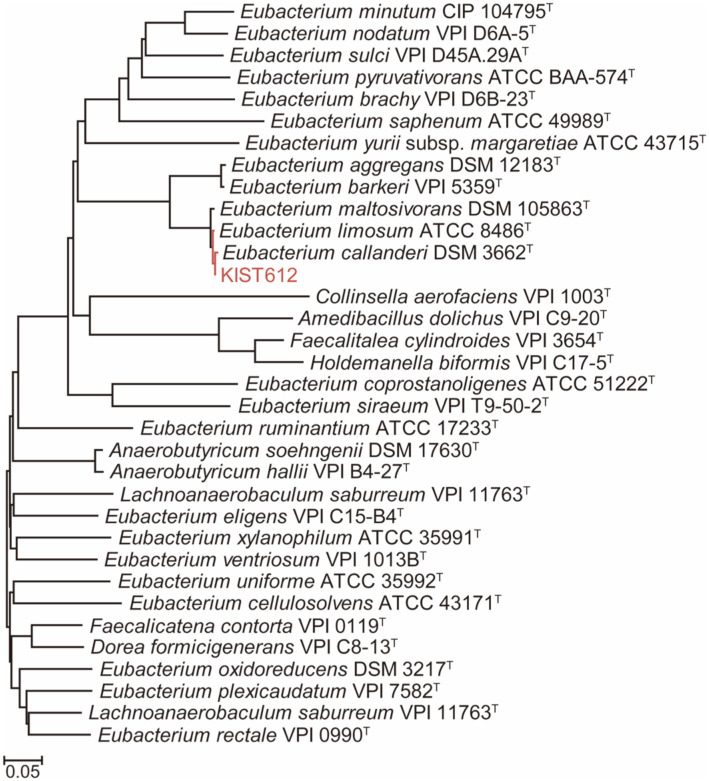
Core-proteome phylogeny of *Eubacterium* spp. and their relatives. The bar means 0.05 nucleotide substitution per site.

The 16S rDNA phylogeny showed that strain KIST612 belongs to the same clade as *E. callanderi* DSM 3662^T^, *E. limosum* ATCC 8486^T^, and *E. maltosivorans* DSM 105863^T^ (Fig. 1). Although *E. aggregans* DSM 12183^T^ and *E. barkeri* VPI 5359^T^ also seem to be closely related with strain KIST612, 16S rDNA identities calculated using BLASTn were lower than the cut-off value (≥ 97%) for the same species (Table 2). According to the updated cut-off threshold (≥ 99%), *Eubacterium* sp. KIST612 could be taxonomically classified as *E. callanderi* sp. or *E. limosum* sp. [26]. Considering the low resolution of the 16S rDNA gene, analyses of the housekeeping genes, coreproteome, ANI and dDDH were additionally performed. As housekeeping genes, we used *rpoB* (RNA polymerase subunit B) and *gyrA* (DNA gyrase subunit A). Clusters of each strain were identified through a housekeeping gene phylogenetic tree, and DNA sequence identities between housekeeping genes were compared using the BLASTn algorithm (http://blast.ncbi.nlm.nih.gov/Blast.cgi). In the phylogenetic tree based on the two housekeeping genes, as in the previous 16S rDNA phylogenetic tree, KIST612 was shown to belong to the same cluster as *E. callanderi* DSM 3662^T^, *E. limosum* ATCC 8486^T^ and *E. maltosivorans* DSM 105863^T^ (Fig. 2). The DNA sequence identity of KIST612 with *rpoB* was 100.0% for *E. callanderi* DSM 3662^T^, 96.0% for *E. limosum* ATCC 8486^T^, and 94.9% for *E. maltosivorans* DSM 105863^T^. In addition, the DNA sequence identity of strain KIST612 with *gyrA* was 100.0% for *E. callanderi* DSM 3662^T^, 95.8% for *E. limosum* ATCC 8486^T^, and 92.8% for *E. maltosivorans* DSM 105863^T^. Therefore, 16S rDNA and housekeeping gene analyses provided a basis for reclassification of KIST612 as *E. callanderi* rather than *E. limosum*, which is the existing classification. The results of our core-proteome analysis provide further supporting information. For core-proteome analysis, 920 to 6,699 proteins (median 2,529) were used, of which a total of 705 core-proteomes were identified. As a result, KIST612 was clustered with *E. callanderi* DSM 3662^T^, *E. limosum* ATCC 8486^T^, and *E. maltosivorans* DSM 105863^T^, as shown by 16S rDNA and housekeeping phylogeny (Fig. 3). Moreover, high similarity between the core-proteome of KIST612 and *E. callanderi* DSM 3662^T^ was also shown.

### Sequence Similarity between *Eubacterium* spp. Based on Average Nucleotide Identity (ANI) and Digital DNA-DNA Hybridization (dDDH)

**Fig. 4:**
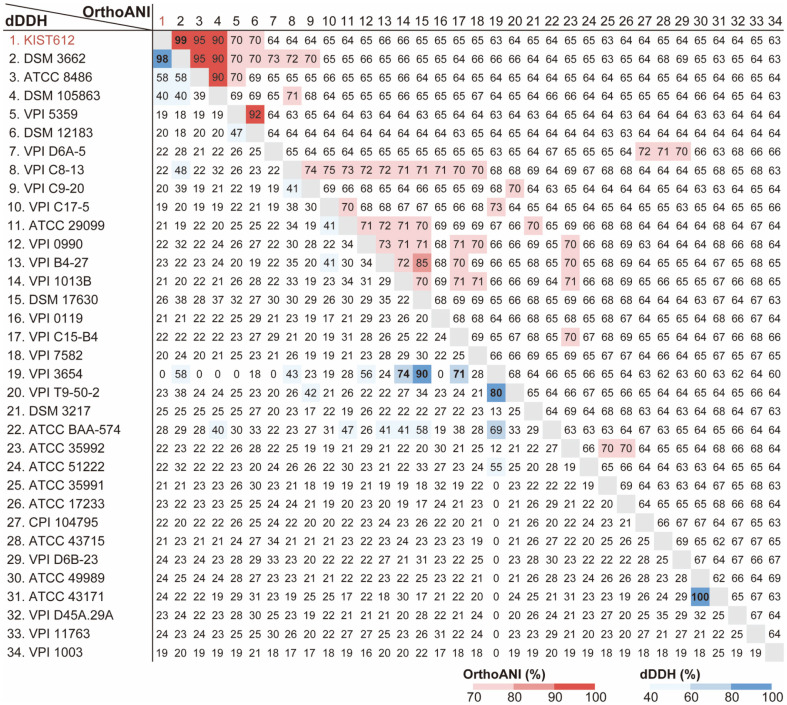
Genomic metrics of *Eubacterium* spp. and their relatives. Bold text indicates values above the species threshold (ANI ≥ 96%; dDDH ≥ 70%).

In general, when the identity between genomes through ANI is ≥ 96%, they are considered as belonging to the same species. The OrthoANIu and dDDH results on the genome sequences between *E. callanderi* DSM 3662^T^, *E. limosum* ATCC 8486^T^, and *E. maltosivorans* DSM 105863^T^ confirmed them to be clustered with KIST612 in 16S rDNA and housekeeping genes. As a result, OrthoANIu values showed similarities of 99.8% for *E. callanderi* DSM 3662^T^, 94.6% for *E. limosum* ATCC 8486^T^, and 89.7% for *E. maltosivorans* DSM 105863^T^ with KIST612 (Fig. 4). All three are above the overall average OrthoANIu value of 67.6%, but only KIST612 strain and *E. callanderi* DSM 3662^T^ returned values of ≥ 96% and are considered in the same species. Similar to OrthoANI, dDDH was used to compare *E. callanderi* DSM 3662^T^, *E. limosum* ATCC 8486^T^, and *E. maltosivorans* DSM 105863^T^ with KIST612 strain. The dDDH results were calculated at 98.4% similarity for *E. callanderi* DSM 3662^T^, 57.8% for *E. limosum* ATCC 8486^T^, and 39.5% for *E. maltosivorans* DSM 105863^T^ with KIST612. All three showed higher than the overall average dDDH value of 25.3%, but among them, KIST612 and *E. callanderi* DSM 3662^T^ are considered to be the most similar species. As a result of our combined analysis by OrthoANIu and dDDH, KIST612 was most likely derived from *E. callanderi*.

### Comparison of Metabolic Pathways between *E. limosum* ATCC 8486^T^, *E. callanderi* DSM 3662^T^, and Strain KIST612

**Fig. 5:**
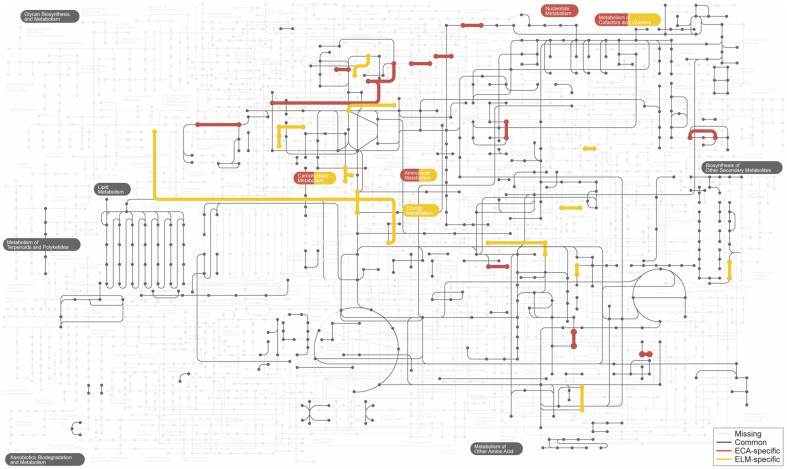
Comparison of metabolic pathways between two *E. callanderi* spp. and *E. limosum* ATCC 8486^T^ using iPath3. ECA: *E. callanderi*; ELM: *E. limosum*.

We found several differences between *E. limosum* ATCC 8486^T^ and the two *E. callanderi* strains, whereas the same pathway was observed in KIST612 and *E. callanderi* DSM 3662^T^. The two species had common proteins involved in lipid metabolism and secondary metabolite biosynthesis. However, they showed differences in their central pathways, including carbohydrate metabolism, amino acid metabolism, energy metabolism, nucleotide metabolism, cofactor metabolism, and vitamin metabolism (Fig. 5). Several proteins were only encoded in *E. callanderi*. For example, 4-α-glucanotransferase, glucose-1-phosphate cytidylyltransferase, diacylglycerol kinase, and gluconolactonase were found, indicating the adaptability of KIST612 under heterotrophic conditions. In addition, we found phosphoribosylglycinamide formyltransferase 1 and aminomethyltransferase in the genome sequences of *E. callanderi*. The two enzymes catalyze the formation of 10-formyltetrahydrofolate and 5,10-methenyl/methylenetetrahydrofolate, respectively. Considering that 10-formyltetrahydrofolate, 5,10-methenyltetrahydrofolate, and 5,10-methylenetetrahydrofolate are intermediates of the Wood-Ljungdahl pathway, which is key metabolism for C1 gas utilization, it could be inferred that *E. callanderi* may more easily uptake CO_2_ and CO than *E. limosum* ATCC 8486^T^. This may also cause the difference in CO tolerance and growth rate under CO condition between the two strains [1-3]. Additionally, the slower metabolic flux of *E. limosum* ATCC 8486^T^ compared to KIST612 may be behind the accumulation of reducing equivalents, such as NADH, which leads to the butyrate production of ATCC 8486^T^ in long-term culture under the H_2_/CO_2_ condition [4].

### Description of *Eubacterium* callanderi KIST612

In the study, we propose reclassification of strain KIST612 from *E. limosum* to *E. callanderi* based on substantial evidence. *E. callanderi* KIST612 is a gram-positive bacterium that is rod-shaped and does not form spores. It was isolated from an anaerobic digester as an obligate anaerobic strain and can utilize various C1 carbon sources, including CO_2_ (with H_2_), CO, and methanol (with CO_2_). KIST612 can optimally grow at 37°C and pH 6.0-7.2. Chang *et al*. (1997) presented its detailed physiological characteristics. This strain is noteworthy for its ability to produce butyrate, making it a potentially valuable resource for large-scale industrial processes in the energy field. *E. callanderi* KIST612 is also known for its high tolerance to CO (until 2 atm), which is uncommon among strains that utilize CO as a substrate. The adaptability of *E. callanderi* KIST612 to heterotrophic conditions has also been confirmed. Despite some missing information on KIST612, the strain could be predicted based on the description of *E. callanderi* DSM 3662^T^ [27]. The peptidoglycan from the cell wall of KIST612 may contain muramic acid, glucosamine, lysine, ornithine, serine, glutamic acid, and alanine. Additionally, the strain may utilize several methoxylated aromatic substrates such as ferulate, sinapate, syringate, vaniliate, 3,4,5-trimethoxycinnamate, and vanillin.

## Figures and Tables

**Table 1:** Genome information used in the study.

Strain	Accession number	Size (Mbp)	Contig	G+C (mol%)	CDS
***Eubacterium* sp. KIST612**	**GCF_000152245**	**4.3**	**1**	**47**	**4,579**
*Am. dolichus* VPI C9-20^T^	GCF_000154285	2.2	25	38	2,139
*An. hallii* VPI B4-27^T^	GCF_000173975	3.3	175	38	2,798
*An. soehngenii* DSM 17630^T^	GCF_009697165	3.2	69	38	2,700
*C. aerofaciens* VPI 1003^T^	GCF_000169035	2.4	25	61	2,096
*D. formicigenerans* VPI C8-13^T^	GCF_025150245	3.2	1	41	2,988
*E. aggregans* DSM 12183^T^	GCF_900107815	2.8	66	49	2,699
*E. barkeri* VPI 5359^T^	GCF_900107125	3.0	46	48	2,739
*E. brachy* VPI D6B-23^T^	GCA_000488855	1.5	27	38	1,358
*E. callanderi* DSM 3662^T^	GCF_900142645	4.4	34	47	4,148
*E. cellulosolvens* ATCC 43171^T^	GCF_000621585	3.3	87	49	2,694
*E. coprostanoligenes* ATCC 51222^T^	GCF_900167205	1.8	36	40	1,641
*E. eligens* VPI C15-B4^T^	GCF_000146185	2.8	3	38	2,621
*E. limosum* ATCC 8486^T^	GCF_000807675	4.4	1	47	4,073
*E. maltosivorans* DSM 105863^T^	GCF_002441855	4.3	1	48	3,979
*E. minutum* CIP 104795^T^	GCA_003433305	1.9	124	46	1,516
*E. nodatum* VPI D6A-5^T^	GCA_000510425	1.8	29	38	1,569
*E. oxidoreducens* DSM 3217^T^	GCF_900104415	2.9	37	40	2,581
*E. plexicaudatum* VPI 7582^T^	GCF_000364225	6.7	6	43	6,699
*E. pyruvativorans* ATCC BAA-574^T^	GCF_900102225	2.2	58	55	1,864
*E. ramulus* ATCC 29099^T^	GCF_000469345	3.4	227	43	3,061
*E. rectale* VPI 0090^T^	GCA_000020615	3.4	1	41	3,161
*E. ruminantium* ATCC 17233^T^	GCF_900167085	2.8	37	37	2,461
*E. saphenum* ATCC 49989^T^	GCA_000161975	1.1	5	41	920
*E. siraeum* VPI T9-50-2^T^	GCA_000382085	2.7	51	45	2,305
*E. sulci* VPI D45A.29A^T^	GCF_001189495	1.7	1	40	1,579
*E. uniforme* ATCC 35992^T^	GCF_900167115	2.9	47	32	2,386
*E. ventriosum* VPI 1013B^T^	GCF_000153885	2.9	38	35	2,476
*E. xylanophilum* ATCC 35991^T^	GCF_000518685	2.6	55	40	2,151
*E. yurii* subsp. *margaretiae* ATCC 43715^T^	GCA_000146855	2.5	84	32	2,134
*F. contorta* VPI 0119^T^	GCF_902375555	5.2	13	46	4,425
*F. cylindroides* VPI 3654^T^	GCF_000469305	1.9	143	35	1,930
*H. biformis* VPI C17-5^T^	GCF_000156655	2.5	161	34	2,359
*L. saburreum* VPI 11763^T^	GCF_000185385	3.1	150	36	2,724

**Table 2:** Sequence identities of 16S rDNA and housekeeping genes of *Eubacterium* spp. with strain KIST612.

Strain	Identity (%)		
	16S rDNA	*rpoB*	*gyrA*
*E. callanderi* DSM 3662^T^	99.8	100.0	100.0
*E. limosum* ATCC 8486^T^	99.2	96.0	95.8
*E. maltosivorans* DSM 105863^T^	97.3	94.9	92.8
*E. aggregans* DSM 12183^T^	94.4	78.5	76.6
*E. barkeri* VPI 5359^T^	94.3	–^a^	76.7

^a^No significant similarity
